# Whole-Genome Draft Assemblies of Difficult-to-Classify Escherichia coli O157 and Non-O157 Isolates from Feces of Canadian Feedlot Cattle

**DOI:** 10.1128/MRA.00168-20

**Published:** 2020-04-09

**Authors:** Vinicius Silva Castro, Eduardo Eustáquio de Souza Figueiredo, Tim McAllister, Robin King, Tim Reuter, Rodrigo Ortega Polo, Carlos Adam Conte, Kim Stanford

**Affiliations:** a Institute of Chemistry, Federal University of Rio de Janeiro, Rio de Janeiro, Brazil; b Department of Food and Nutrition, Federal University of Mato Grosso, Cuiaba, Brazil; c Agriculture and Agri-Food Canada, Lethbridge Research and Development Centre, Lethbridge, Canada; d Alberta Agriculture and Forestry, Edmonton, Canada; e Alberta Agriculture and Forestry, Agriculture Centre, Lethbridge, Canada; University of Maryland School of Medicine

## Abstract

Forty-eight Escherichia coli strains were chosen due to variable detection of *stx* or serogroup by PCR. Although all strains were initially determined to be Shiga toxin-producing Escherichia coli (STEC), their genomes revealed 11 isolates carrying *stx*_1a_, *stx*_1b_, *stx*_2a_, and/or *stx*_2b_. Assembled genome sizes varied between 4,667,418 and 5,556,121 bp, with *N*_50_ values between 79,648 and 294,166 bp and G+C contents between 50.3% and 51.4%.

## Abstract (precis)

Forty-eight Escherichia coli strains were chosen due to variable detection of *stx* or serogroup by PCR. Although all strains were initially determined to be Shiga toxin-producing Escherichia coli (STEC), their genomes revealed 11 isolates carrying *stx*_1a_, *stx*_1b_, *stx*_2a_, and/or *stx*_2b_. Assembled genome sizes varied between 4,667,418 and 5,556,121 bp, with *N*_50_ values between 79,648 and 294,166 bp and G+C contents between 50.

## ANNOUNCEMENT

Escherichia coli bacteria are ubiquitous microorganisms that are most often commensals, but some groups, such as Shiga toxin-producing E. coli (STEC), possess genes that confer pathogenicity in humans, leading to vomiting, bloody diarrhea, and hemolytic uremic syndrome (1, 2). Ruminants are the main reservoir of STEC (3, 4), but STEC may also be present in other vectors, including birds, rodents, flies, and water (5–8). Shedding of STEC in cattle appears to be influenced by season, housing density, and the host (9, 10). In this project, 48 E. coli strains were selected based on consistent or inconsistent detection of *stx* and/or serogroup by PCR. (9). Within our collection of approximately 15,000 E. coli strains, relatively few, other than those selected for whole-genome sequencing (WGS), have had inconsistent PCR results. Thirty-one strains were consistent for serogroup detection by PCR, and they were confirmed by WGS (Table 1) using the E. coli O and H group (EcOH) database in ABRicate version 0.8.10 (https://github.com/tseemann/ABRICATE) (11). All 48 strains were classified as STEC by PCR based on carriage of *stx*_1_ and/or *stx*_2_. Fourteen strains were consistent for PCR detection of *stx*_1_ and/or *stx*_2_, but only 11 of these were STEC based on WGS. These strains were positive for antimicrobial resistance genes for beta-lactams (100%), tetracycline (81.2%), and sulfonamides (8.3%), as assessed by using the Comprehensive Antibiotic Resistance database (12) and ABRicate. In addition, the virulence profile was analyzed using the ABRicate E. coli_VF database, and strains possessed *stx*_1a_, *stx*_1b_, *stx*_2a_, *stx*_2b_, and adhesion genes (i.e., *toxB*, *fdeC*, *csg*, and variants) related to biofilm formation and several types of secretion systems, including the type III secretion system encoded in the locus of enterocyte effacement (LEE) or non-LEE-encoded type III effector. For all ABRicate analyses, default parameters were used except for minimum DNA percent coverage, which was set to 60%.

**TABLE 1:** Overview of the E. coli draft genome assemblies

Strain	Serotype	BioSample accession no.	SRA accession no.	Assembly accession no.	Total no. of reads	Sequencing depth (×)	G+C content (%)	*N*_50_ (bp)	No. of contigs	Genome size (bp)	No. of CDSs^*j*^	Resistance gene(s); *stx* subtype(s)
CAP01^*g*^	O103:H2	SAMN13870004	SRR10947171	ASM1102935v1	2,436,574	85	51.2	127,894	211	5,114,061	4,883	*arsB*(mob)^*k*^, *bla*_EC-18_; *csg*
CAP02^*a*^^,^^*h*^	O103:H11	SAMN13870005	SRR10947170	ASM1102932v1	2,235,508	75	51.0	93,966	316	5,434,829	5,263	*arsB*(mob), *bla*_EC-18_, *toxB*; *csg*, *stx*_1a_, *stx*_1b_
CAP03^*a*^^,^^*h*^	O157:H7	SAMN13870006	SRR10947160	ASM1102933v1	2,375,528	82	51.0	142,962	216	5,380,304	5,104	*arsB*(mob), *bla*_EC-15_, *toxB*; *csg*, *stx*_1a_, *stx*_1b_, *stx*_2a_, *stx*_2b_
CAP04^*a*^^,^^*h*^	O26:H11	SAMN13870007	SRR10947159	ASM1102928v1	2,473,316	86	51.1	99,468	292	5,556,121	5,401	*arsB*(mob), *bla*_EC-18_, *toxB*; *csg*, *stx*_1a_*, stx*_1b_
CAP05^*b*^^,^^*i*^	O9:H30	SAMN13870008	SRR10947158	ASM1102924v1	2,257,402	75	51.1	141,506	100	4,802,339	4,457	*aph(3'')-Ib*, *aph(6)-Id*, *arsB*(mob), *bla*_EC-15_, *bla*_TEM-1_, *dfrA5*, *sul2*; *csg*
CAP06^*c*^^,^^*i*^	O110:H30	SAMN13870009	SRR10947157	ASM1103269v1	2,333,812	80	50.9	151,454	123	5,369,514	5,158	*arsB*(mob), *bla*_EC-13_, *tet(A)*; *csg*
CAP08^*h*^	O103:H2	SAMN13870010	SRR10947156	ASM1103268v1	2,612,588	86	51.1	200,865	179	5,230,549	5,032	*arsB*(mob), *bla*_EC-18_; *csg*
CAP09^*d*^	H34	SAMN13870011	SRR10947155	ASM1103265v1	2,478,842	88	51.2	104,339	117	4,782,368	4,456	*arsB*(mob), *bla*_EC_; *csg*
CAP10^*a*^^,^^*h*^	O157:H7	SAMN13870012	SRR10947154	ASM1103264v1	2,161,150	71	50.9	157,234	188	5,285,310	4,978	*arsB*(mob), *bla*_EC-15_, *toxB*; *csg*, *stx*_1a_, *stx*_1b_
CAP11^*g*^	O121:H7	SAMN13870013	SRR10947153	ASM1103262v1	2,173,434	75	51.1	241,028	90	5,016,814	4,700	*arsB*(mob), *bla*_EC-18_; *csg*
CAP12^*c*^^,^^*i*^	O9:H4	SAMN13870014	SRR10947169	ASM1103254v1	2,220,446	76	51.1	83,564	137	4,775,627	4,469	*arsB*(mob), *bla*_EC-18_; *csg*
CAP13^*b*^^,^^*i*^	H:28	SAMN13870015	SRR10947168	ASM1103259v1	2,193,184	73	50.9	216,462	91	5,273,949	4,971	*bla*_EC_; *csg*
CAP14^*g*^	O103:H2	SAMN13870016	SRR10947167	ASM1103258v1	2,238,086	77	51.1	92,301	214	5,156,047	4,965	*arsB*(mob), *bla*_EC-18_; *csg*
CAP15^*i*^	O45:H51	SAMN13870017	SRR10947166	ASM1103252v1	2,443,526	82	51.1	135,724	173	5,184,896	4,990	*arsB*(mob), *bla*_EC-18_, *tet(C)*; *csg*
CAP16^*a*^^,^^*h*^	O26:H11	SAMN13870018	SRR10947165	ASM1103253v1	2,447,720	85	51.2	98,248	312	5,547,588	5,401	*arsB*(mob), *bla*_EC-18_, *toxB*; *csg*, *stx*_1a_, *stx*_1b_
CAP17^*b*^^,^^*i*^	O17:H18	SAMN13870019	SRR10947164	ASM1103244v1	2,220,086	75	50.5	142,741	274	5,463,072	5,174	*aph(3'')-Ib*, *aph(6)-Id*, *arsB*(mob), *bla*_EC-8_, *tet(B)*; *csg*
CAP18^*a*^^,^^*h*^	O145:H28	SAMN13870020	SRR10947163	ASM1103247v1	2,274,708	74	50.3	139,371	220	5,318,832	5,028	*bla*_EC_, *toxB*; *csg*, *stx*_1a_, *stx*_1b_, *stx*_2a_, *stx*_2b_
CAP19^*a*^^,^^*g*^	O121:H7	SAMN13870021	SRR10947162	ASM1103248v1	2,020,850	67	50.6	187,631	114	5,105,071	4,856	*arsB*(mob), *bla*_EC-18_; *csg*, *stx*_1a_, *stx*_1b_
CAP20^*d*^^,^^*i*^	O17:H18	SAMN13870022	SRR10947161	ASM1103242v1	2,506,734	84	50.8	211,387	126	5,137,726	4,774	*arsB*(mob), *bla*_EC-8_; *csg*
CAP21^*d*^^,^^*i*^	O153:H8	SAMN13870023	SRR10958942	ASM1103243v1	1,767,498	65	50.9	252,910	90	5,140,948	4,863	*arsB*(mob), *bla*_EC-18_, *qacG2*, *tet(A)*, *tet(M)*; *csg*
CAP22^*e*^^,^^*i*^	O8:H2	SAMN13870024	SRR10958941	ASM1103235v1	2,055,856	66	50.9	101,879	169	5,377,305	5,056	*arsB*(mob), *bla*_EC-18_; *csg*
CAP23^*a*^^,^^*h*^	O145:H28	SAMN13870025	SRR10958930	ASM1103234v1	1,975,732	68	50.9	213,717	222	5,315,836	5,033	*bla*_EC_, *toxB*; *csg*, *stx*_1a_, *stx*_1b_
CAP24^*e*^^,^^*i*^	O76:H34	SAMN13870026	SRR10958920	ASM1103236v1	2,383,008	82	50.9	119,506	108	4,796,204	4,483	*aadA2*, *bla*_EC_, *dfrA12*, *qacEdelta1*, *sul1*, *tet(A)*; *csg*
CAP25^*g*^	O121:H7	SAMN13870027	SRR10958919	ASM1103232v1	2,381,330	86	51.2	185,921	115	5,149,544	4,862	*arsB*(mob), *bla*_EC-18_; *csg*
CAP26^*g*^	O45:H11	SAMN13870028	SRR10958918	ASM1103233v1	2,021,072	71	51.1	109,224	124	4,989,320	4,688	*arsB*(mob), *bla*_EC-13_; *csg*
CAP27^*g*^	O103:H8	SAMN13870029	SRR10958917	ASM1103228v1	1,971,998	70	51.0	128,935	187	5,300,501	4,991	*arsB*(mob), *bla*_EC-18_; *csg*
CAP28^*d*^^,^^*i*^	O5:H32	SAMN13870030	SRR10958916	ASM1103226v1	1,985,702	72	51.3	79,648	172	5,386,841	5,141	*arsB*(mob), *bla*_EC-15_, *tet(B)*; *csg*
CAP29^*d*^^,^^*i*^	O5:H19	SAMN13870031	SRR10958915	ASM1103227v1	2,525,640	82	51.1	189,310	98	5,252,991	5,053	*arsB*(mob), *bla*_EC-18_, *qacG2*, *tet(A)*, *tet(M)*; *csg*
CAP30^*f*^^,^^*i*^	H34	SAMN13870032	SRR10958914	ASM1103223v1	1,561,088	56	51.1	205,590	57	4,718,264	4,362	*arsB*(mob), *bla*_EC-15_; *csg*
CAP31^*g*^	O157:H29	SAMN13870033	SRR10958940	ASM1103222v1	1,736,606	60	50.7	142,303	140	4,948,575	4,579	*arsB*(mob), *bla*_EC-15_, tet(C); *csg*
CAP32^*a*^^,^^*g*^	O145:H28	SAMN13870034	SRR10958939	ASM1103218v1	2,050,004	74	51.3	140,944	251	5,265,290	4,997	*bla*_EC_, *toxB*; *csg*, *stx*_1a_, *stx*_1b_
CAP33^*a*^^,^^*h*^	O103:H25	SAMN13870035	SRR10958938	ASM1103219v1	2,546,878	82	51.2	103,076	250	5,325,616	5,150	*arsB*(mob), *bla*_EC-18_; *csg*, *stx*_1a_, *stx*_1b_
CAP34^*b*^^,^^*i*^	O8:H10	SAMN13870036	SRR10958937	ASM1103216v1	2,462,412	82	50.7	116,765	125	4,963,326	4,722	*aadA1*, *aph(3'')-Ib*, *aph(6)-Id*, *arsB*(mob) *bla*_EC_, *bla*_TEM-1_, *floR*, *sul2*; *csg*
CAP35^*h*^	O45:H45	SAMN13870037	SRR10958936	ASM1103213v1	1,886,694	61	50.9	85,497	183	5,124,512	4,916	*arsB*(mob), *bla*_EC-15_, *tet(C)*; *csg*
CAP36^*g*^	O26:H9	SAMN13870038	SRR10958935	ASM1103212v1	2,219,820	73	51.4	150,844	99	4,745,341	4,414	*arsB*(mob), *bla*_EC_; *csg*
CAP37^*d*^^,^^*i*^	O187:H52	SAMN13870039	SRR10958934	ASM1103205v1	1,980,034	64	51.2	215,461	100	4,933,181	4,634	*arsB*(mob), *bla*_EC-18_; *csg*
CAP38^*g*^	O157:H29	SAMN13870040	SRR10958933	ASM1103204v1	1,834,588	59	50.4	186,647	113	5,066,397	4,714	*arsB*(mob), *bla*_EC-15_, *tet(A)*; *csg*
CAP39^*g*^	O45:H4	SAMN13870041	SRR10958932	ASM1103209v1	1,705,628	54	51.1	161,396	126	4,971,004	4,633	*aph(3'')-Ib*, *aph(6)-Id*, *arsB*(mob), *bla*_EC-18_, *floR*, *sul2*, *tet(A)*; *csg*
CAP40^*f*^^,^^*i*^	O53:H32	SAMN13870042	SRR10958931	ASM1103207v1	2,552,634	81	51.2	147,707	80	4,667,418	4,308	*arsB*(mob), *bla*_EC-15_; *csg*
CAP41^*g*^	O103:H19	SAMN13870043	SRR10958929	ASM1103202v1	2,058,560	65	50.9	203,947	56	4,774,483	4,398	*arsB*(mob), *bla*_EC-18_; *csg*
CAP42^*g*^	O26:H32	SAMN13870044	SRR10958928	ASM1102813v1	2,590,136	84	51.0	294,166	59	4,774,449	4,441	*arsB*(mob), *bla*_EC_; *csg*
CAP43^*f*^^,^^*i*^	O51:H14	SAMN13870045	SRR10958927	ASM1102814v1	2,378,144	74	50.8	132,565	104	5,133,685	4,729	*arsB*(mob), *bla*_EC_; *csg*
CAP44^*g*^	O45:H38	SAMN13870046	SRR10958926	ASM1102812v1	2,140,178	69	51.0	252,725	89	4,953,772	4,583	*arsB*(mob), *bla*_EC-18_; *csg*
CAP45^*g*^	O157:H12	SAMN13870047	SRR10958925	ASM1102810v1	1,790,792	57	51.2	213,375	81	4,764,211	4,517	*arsB*(mob), *bla*_EC_; *csg*
CAP46^*g*^	O103:H21	SAMN13870048	SRR10958924	ASM1102808v1	2,181,136	68	51.2	165,437	110	5,031,905	4,705	*arsB*(mob), *bla*_EC-18_; *csg*
CAP47^*a*^^,^^*g*^	O145:H28	SAMN13870049	SRR10958923	ASM1102805v1	2,181,386	73	51.0	147,767	222	5,280,428	5,009	*bla*_EC_; *csg*, *stx*_1a_, *stx*_1b_
CAP48^*g*^	O157:H38	SAMN13870050	SRR10958922	ASM1102802v1	3,040,268	102	50.8	120,842	151	5,340,491	5,101	*arsB*(mob), *bla*_EC-18_; *csg*
CAP49^*g*^	O103:H14	SAMN13870051	SRR10958921	ASM1102804v1	1,353,030	47	50.7	122,791	243	5,458,338	5,263	*arsB*(mob), *bla*_EC-18_; *csg*

a Strains confirmed as STEC by WGS.

b Formerly identified as O26 by PCR.

c Formerly identified as O45 by PCR.

d Formerly identified as O103 by PCR.

e Formerly identified as O145 by PCR.

f Formerly identified as O157 by PCR.

g Consistent serogroup by PCR and inconsistent virulence factors.

h Consistent serogroup and virulence factors by PCR.

i Inconsistent serogroup and virulence factors by PCR.

j CDS, coding DNA sequences.

k *arsB*(mob), mobile version of *arsB*.

The isolation was performed as described by Stanford et al. (9). Briefly, fecal aliquots were enriched in E. coli broth (EMD Millipore, Darmstadt, Germany) (6 h at 37°C) and then subjected to immunomagnetic separation using RapidChek Confirm STEC kits (Romer Labs Technology, Inc., Newark, DE, USA) and magnetic-bead separation using Pickpen (BioControl Systems, Bellevue, WA, USA). The bead-bacteria mixture was then plated on MacConkey agar (Dalynn Biologicals, Calgary, Canada) and incubated (18 to 24 h at 37°C). Three to nine sorbitol-negative colonies/plate were subjected to PCR screening for E. coli target genes (13).

For genomic DNA analysis, the methodology was as described by Bumunang et al. (14), where the DNA of isolates was extracted from overnight bacterial cultures grown in 9 ml of Luria-Bertani broth (Merck, Kirkland, Canada) using the ZR fungal/bacterial DNA miniprep kit (Epigenetics Company, Irvine, CA, USA) according to the manufacturer’s instructions. WGS was performed at the Agri-Food Laboratories (Alberta Agriculture and Forestry, Edmonton, Canada). DNA was quality checked and quantified using a Qubit fluorometer (Thermo Fisher, Waltham, MA, USA) and Tapestation 4200 system (Agilent, Santa Clara, CA, USA).

Sequencing was performed on an Illumina MiSeq platform using the MiSeq reagent kit V2 (Illumina, San Diego, CA, USA) to produce 251-bp paired-end reads. Sequencing reads were *de novo* assembled into contigs using the Shovill pipeline v1.0.4 (https://github.com/tseemann/shovill). Shovill included trimming, which was performed with Trimmomatic v0.39, and *de novo* assembly was performed with SPAdes v3.13.1 (15). The quality report for the assembly was measured using QUAST v5.0.0, and draft genome assemblies were annotated with Prokka (16). The assignment of the strain to species was verified using Centrifuge 1.04 (17) and the p-compressed index of 5,202 taxa from the RefSeq database of NCBI. Default parameters were used for these software. Across strains, the assembled genome sizes varied between 4,667,418 and 5,556,121 bp and 56 and 316 contigs, with sequence coverages between 47× and 102×. The *N*_50_ values varied between 79,648 and 294,166 bp, and the G+C contents varied between 50.3% and 51.4%.

### Data availability.

The raw Illumina data (sequence read archive [SRA]) and genome contigs with respective annotations were deposited in NCBI and are described in Table 1. All SRA and genome annotation data were included in the BioProject number PRJNA601484.
